# Personalized allele-specific antisense oligonucleotides for GNAO1-neurodevelopmental disorder

**DOI:** 10.1016/j.omtn.2024.102432

**Published:** 2024-12-22

**Authors:** Inna Shomer, Nofar Mor, Shaul Raviv, Noga Budick-Harmelin, Tanya Matchevich, Sharon Avkin-Nachum, Yoach Rais, Rebecca Haffner-Krausz, Ariela Haimovich, Aviv Ziv, Reut Fluss, Bruria Ben-Ze’ev, Gali Heimer, Denis N. Silachev, Vladimir L. Katanaev, Dan Dominissini

**Affiliations:** 1 Cancer Research Center and Wohl Institute for Translational Medicine, Tel Hashomer, Ramat Gan, Israel; 2 Department of Veterinary Resources, Weizmann Institute of Science, Rehovot, Israel; 3 Sheba Medical Center, Edmond and Lilly Safra Children’s Hospital, Tel Hashomer, Israel; 4 Institute of Life Sciences and Biomedicine, Far Eastern Federal University, 690090 Vladivostok, Russia; 5 A.N. Belozersky Research Institute of Physico-Chemical Biology, Moscow State University, 119992 Moscow, Russia; 6 Department of Cell Physiology and Metabolism, Faculty of Medicine, Translational Research Center in Oncohaematology, University of Geneva, 1211 Geneva, Switzerland; 7 Faculty of Medicine, Tel Aviv University, Tel Aviv, Israel

**Keywords:** MT: Oligonucleotides: Therapies and Applications, GNAO1, personalized ASOs, antisense oligonucleotides, ASOs, individualized ASOs, cellular models, mouse model, E246K, allele-specific ASOs

## Abstract

GNAO1-associated disorders are ultra-rare autosomal dominant conditions, which can manifest, depending on the exact pathogenic variant in *GNAO1*, as a spectrum of neurological phenotypes, including epileptic encephalopathy, developmental delay with movement disorders, or late-onset dystonia. There are currently no effective treatments available, apart from symptomatic options. In this work, we suggest harnessing personalized RNA therapy to treat *GNAO1* patients and focus specifically on a recurrent pathogenic variant (E246K). We systemically screened allele-specific antisense oligonucleotides (ASOs) targeting the mutated allele to identify a potent and specific sequence using both reporter-based platforms and a patient-derived cellular model. We show that reduction of mutated *GNAO1 in vitro* by knockout or by ASO has a beneficial functional outcome, which can be measured by cAMP accumulation and gene expression changes. We established a Gnao1-E246K mouse model that shows a neurological phenotype, which partially recapitulates the human condition. Due to sequence similarity, the mouse can be treated with the selected ASO to test treatment efficacy in animal models, as shown *in vitro* using murine neural progenitor cells. Our results demonstrate a beneficial effect for the reduction of mutated *GNAO1* by ASO in patient-derived models, demonstrating its feasibility as a therapeutic approach.

## Graphical abstract

**Figure:**
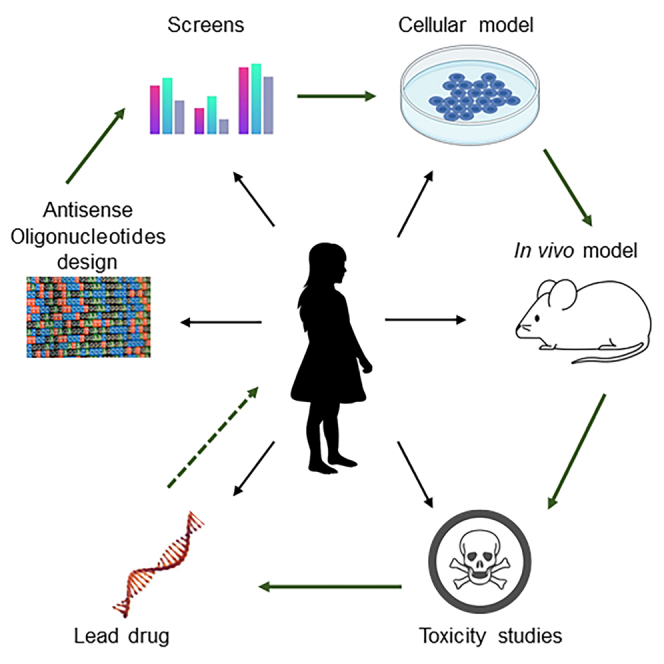

## Teaser

Shomer and colleagues describe a potential beneficial effect for allele-specific antisense oligonucleotides (ASOs) targeting the E246K mutation in GNAO1. Tested in patient-derived neuronal cells and cells from the Gnao1-E246K mouse model, these ASOs selectively reduce mutant expression, improving cellular outcomes and supporting the feasibility of personalized RNA therapy for GNAO1 disorders.

## Introduction

The *GNAO1* gene encodes the α subunit of Go, one of the most abundantly expressed membrane-associated proteins in the human central nervous system (CNS), mediating signal transductions from inhibitory and other G protein-coupled receptors.^1^^,^^2^ Multiple functions have been attributed to Gαo,^3^ and its dysfunction due to dominant missense mutations results in movement disorders (with dystonia as a main feature), epileptic encephalopathy, and hypotonia.^4^^,^^5^ Traditionally, *GNAO1*-related pathology is defined as one of two ultra-rare neurodevelopmental disorders, developmental and epileptic encephalopathy 17 (OMIM: 615473) or neurodevelopmental disorder with involuntary movements (OMIM: 617493), both stemming from heterozygote mutations in *GNAO1*.^6^^,^^7^ Recently, a milder phenotype has been added to the spectrum of *GNAO1*-related disorders, with the identification of variants resulting in late-onset and mild dystonia.^8^^,^^9^ Mechanistic studies of the pathogenic *GNAO1* variants showed varying aberrations in guanosine triphosphate (GTP) uptake and hydrolysis, G protein-coupled receptor (GPCR) coupling, cyclic AMP (cAMP) signaling, and cellular localization and interactions.^1^^,^^10^^,^^11^^,^^12^ While loss-of-function and gain-of-function mechanismsinitially were attributed to the pathogenic variants,^1^^,^^4^^,^^5^^,^^13^ recent studies indicate a more complex mechanism of action; while reports regarding *GNAO1* deletions correlate with a milder phenotype,^14^^,^^15^ the more clinically severe mutations are recognized as neomorphic.^12^^,^^16^^,^^17^ *GNAO1* is ubiquitously expressed in the brain, with specific regions highlighted with dramatic importance to GNAO1 manifestations, including the striatum^1^ and the motor cortex.^18^ Moreover, clinical improvement is seen in patients with severe movement disorders treated by deep brain stimulation, where electrodes are applied to the globus pallidus internus.^19^

RNA-based therapies are based on chemically modified oligonucleotides, such as antisense oligonucleotides (ASOs) or small interfering RNAs, that can act on essentially every cellular RNA and modulate its abundance, processing, and translational output.^20^ ASOs can manipulate transcript splicing (e.g., nusinersen^21^), block other regulatory sites (STK-001, currently in clinical trials^22^), or efficiently recruit and localize RNase H1 for target RNA degradation^20^^,^^23^ by utilizing the gapmer design. Gapmer ASO structure is composed of two flanking – five 2′-methoxyethyl (2′-MOE)-modified ribonucleotides (RNA) at each terminus and a central region of 2′-deoxynucleotides (DNA). While the flanking 2′-MOE ends prevent nuclease cleavage of the ASO, the chimeric gapmer ASO design directs RNase H1 to the central gap made of DNA, where it performs specific target RNA degradation, as RNase H1 shows high specificity for DNA-RNA duplexes. These modifications, along with phosphorothioate (PS) in the backbone, were used successfully in several US Food and Drug Administration-approved drugs such as inotersen, mipomersen, and volanesorsen to silence target RNAs.^24^

Here, we report the development and optimization of allele-specific ASO as a potential personalized treatment for a patient suffering from a dominant pathogenic variant in *GNAO1*. We employ patient-derived cellular models to test allele-specific gapmer ASOs for selectivity and potency and show that lead candidates can selectively alter *GNAO1* mutated allele expression in both induced pluripotent stem cells (iPSCs) and patient-derived dorsal root ganglion (DRG) neurons. Furthermore, we describe a mouse model established in our lab that harbors the corresponding variant in *mGnao1*, and we present data indicating a partial recapitulation of the patient’s symptoms.

## Results

### Establishing GNAO1-E246K patient-derived cellular model

**Figure 1:**
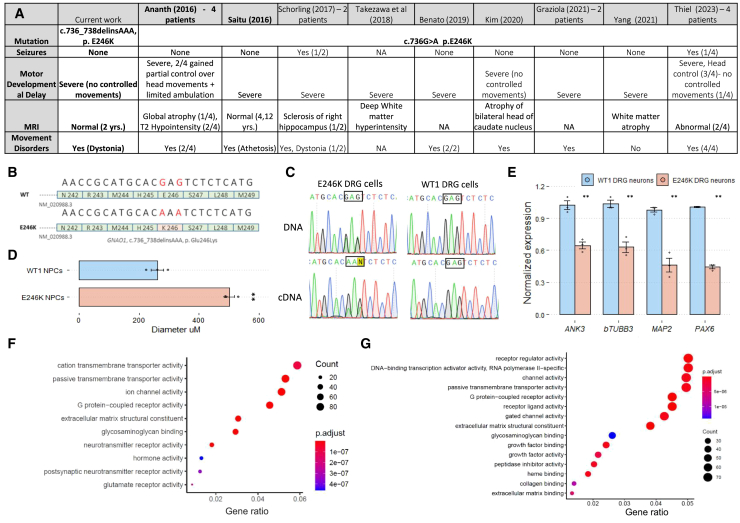
GNAO1-E246K patient-derived cellular model (A) Clinical summary of GNAO1-E246K reported patients, compared to the patient presented in the current work. (B) The patient’s unique *GNAO1* genomic sequence includes two single nucleotide variants, one pathogenic and one synonymous (both marked in red). (C) Chromatograms obtained by Sanger sequencing of gDNA and cDNA, derived from WT1 and patient (E246K) DRG neurons, showing decreased levels of E246K vs. WT transcript of *GNAO1* in patient-derived DRG. Location of mutations are marked by squares. (D) Comparison of average NPC sphere diameters (μM), between WT1 and patient (E246K) cell lines; 3 independent experiments; *n* = 6 NPCs each (∗∗*p* < 0.001). (E) qPCR analysis of transcript levels for differentiation markers in WT1 vs. patient (E246K) DRG neurons (∗∗*p* < 0.001). Expression normalized to *GAPDH*; *n* = 3 (independent experiments). (F) GO enrichment (molecular function) for significantly enriched genes in WT1 vs. patient-derived NPCs. (G) GO enrichment (molecular function) for significantly enriched genes in WT1 vs. patient-derived DRG neurons.

The patient is a 6-year-old female with a *de novo* heterozygote E246K pathogenic variant in *GNAO1*, discovered by exome sequencing. She suffers from severe developmental delay, hypotonia, hyperexcitability, and sleep disorders. She began experiencing movement disorders, including dystonia, at age 5 years, and her brain MRI does not show any pathological findings. While E246K is a recurrent pathogenic variant, previously reported in other patients^25^^,^^26^^,^^27^^,^^28^^,^^29^ (Figure 1A), our patient’s genomic sequence is unique, as it harbors an additional synonymous sequence variation in close proximity to the pathogenic mutated base (*GNAO1*, c.736_738delinsAAA) (Figures 1B and 1C). GTP uptake and hydrolysis analyses show that E246K results in faster GTP uptake compared to wild type (WT) and reduced GTP hydrolysis.^12^ These two changes led to a constitutive GTP-binding state of the G protein, defining E246K as a neomorph,^12^ corresponding with its clinical outcome and previous biochemical analysis.^4^^,^^5^

To further characterize the patient’s condition *in vitro*, we set out to establish a patient-derived cellular model. To that end, we generated iPSCs by reprogramming fibroblasts derived from the patient’s skin-punch biopsy.^30^ As *GNAO1* is ubiquitously expressed in the CNS, we next differentiated the cells to the neural lineage, namely to neural progenitor cells (NPCs) and DRG neurons, using a previously published protocol^31^ (Figures S1A and S1B). Downregulation of pluripotency factors and upregulation of neural markers were confirmed by qPCR, RNA-seq, and immunofluoresence to ensure successful differentiation (Figures S1C–S1E). Patient-derived iPSCs, NPCs, and DRG neurons were then characterized and compared to WT1 cells, which were reprogrammed from healthy donor-derived fibroblasts. A minor decrease was observed in E246K transcript levels compared to WT transcripts, using Sanger sequencing of patient-derived DRG neurons (Figures 1C, S1G, and S1H), possibly resulting from reduced mutated allele expression or stability, as previously described.^5^ Although subjected to the same neural differentiation protocol, patient-derived NPCs and neurons showed accelerated proliferation compared to WT cells, indicated by increased Ki67 staining in neurons (Figure S1F), and NPC sphere size (Figure 1D), which was significantly larger in the patient’s cells (*p* < 0.001). Furthermore, patient iPSCs repeatedly failed to upregulate neural markers (*MAP2*, *NSE*, *PAX6*, *bTUBB3*) to the same levels as observed in WT1 cells (Figure 1E), suggesting aberrant or inadequate differentiation, as seen in additional patient-derived iPSC acquired from GNAO1 patients with a different pathogenic variant (G203R).^32^ Next, RNA-seq data were employed to identify transcriptional differences between patient *in vitro* differentiated NPCs and WT NPCs. In agreement with results obtained by Sanger sequencing, *GNAO1*-targeted analysis in both NPCs and DRG neurons revealed a lower expression of the mutated allele compared to the WT allele of the patient (Figures S1G and S1H). Gene Ontology (GO) enrichment analysis (molecular function) for differentially expressed genes show multiple affected pathways—for example, neurotransmitter activity, hormone activity, and signaling pathways, including GPCR activity (Figures 1F and 1G).

### GNAO1 heterozygote knockout can rescue the aberrant cellular phenotype

**Figure 2:**
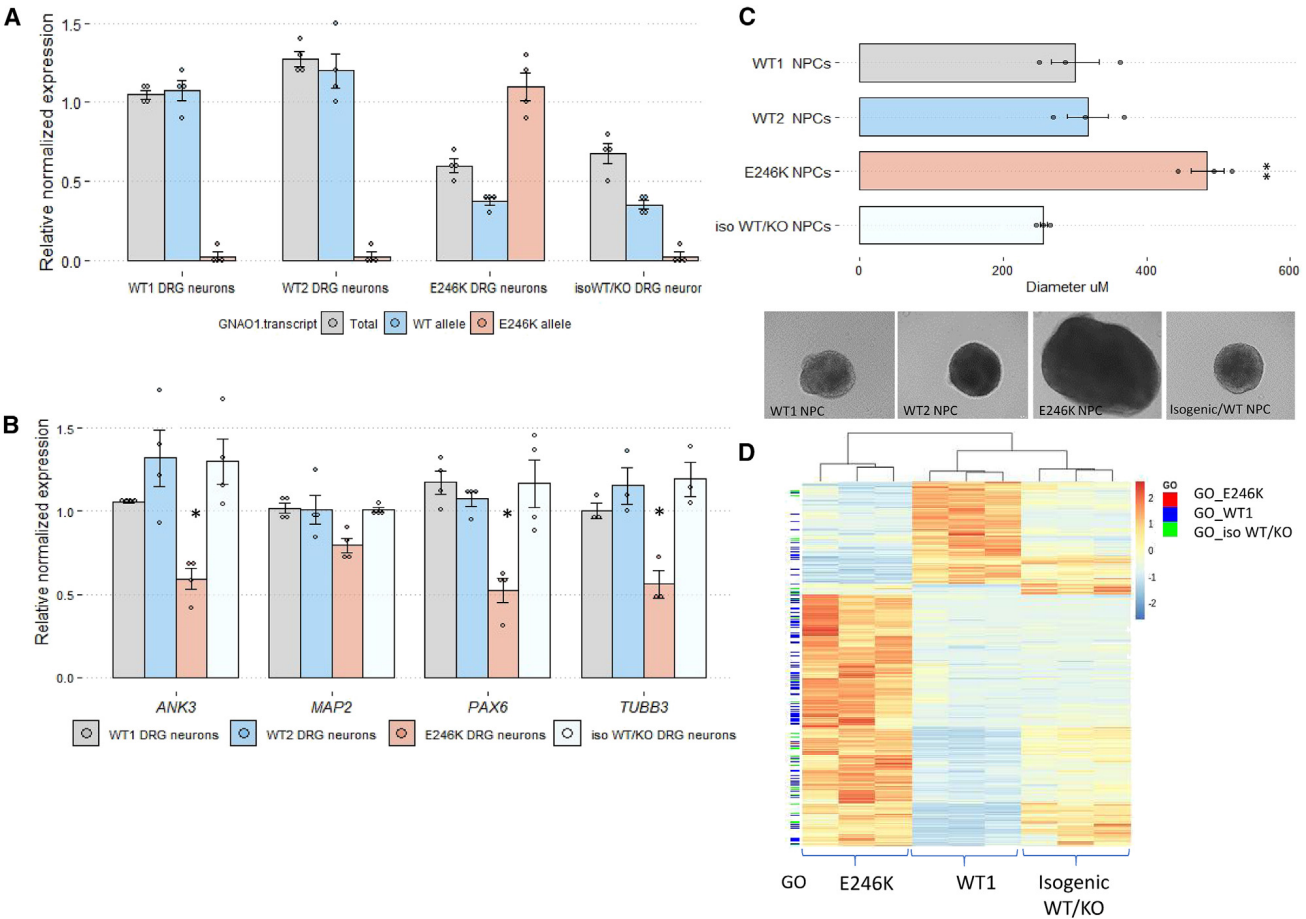
Knockdown of mutant GNAO1 allele in heterozygote can rescue aberrant phenotype (A) Transcript level for total *GNAO1* and, WT/E246K *GNAO1* alleles in WT1, WT2, E246K, and isogenic WT/KO iPSC-derived DRG neurons. E246K allele expression is relative to expression in E246K DRG neurons; total and WT transcript levels are relative to expression in WT1 DRG neurons, normalized to *GAPDH*; *n* = 4. (B) qPCR of neuronal differentiation markers *(MAP2*, *PAX6*, *TUBB3*, and *ANK3)* in WT1, WT2, patient (E246K), and isogenic WT/KO iPSC-derived DRG neurons, normalized to *GAPDH*; *n* = 4 (∗*p* < 0.01). (C) WT1, WT2, patient (E246K), and isogenic WT/KO NPC spheres’ diameters (μM). Three independent experiments; *n* = 6 NPCs each; ∗∗ *p* < 0.001. (D) Heatmap of differentially expressed genes based on RNA-seq for WT1, isogenic (WT/KO), and patient-derived DRG neurons.

We hypothesized that allele-specific silencing of the mutated allele can rescue the cellular phenotype described *in vitro*. Our theory was supported by sporadic publications describing a milder phenotype in patients with *GNAO1* deletion (whole-gene deletion or nonsense variants), including slowly progressive or late-onset dystonic features, with no developmental delays.^8^^,^^14^^,^^33^^,^^34^^,^^35^ We were also encouraged by mouse models with *mGnao1* heterozygote deletion, which were described as viable and fertile,^36^ although one model exhibited neurological manifestations.^37^ To test our hypothesis, we established an isogenic knockout (KO) cell line by targeting the mutated allele in patient-derived iPSCs. We performed knockin by CRISPR-Cas9 and introduced a heterozygote splicing mutation (in addition to the pathogenic variant correction) in the originally mutated allele, resulting in nonsense-mediated decay and KO (referred to as GNAO1^iso-WT/KO^) (Figures S2A and S2B). Expression of *GNAO1* only from the original WT allele and not from the mutated allele was confirmed by qPCR (Figure 2A), resulting in an overall lower expression of *GNAO1* in *GNAO1*^*iso-WT/KO*^ DRG neurons, when compared to WT1 and WT2 DRG neurons. We then subjected WT1, WT2, *GNAO1*^*iso-WT/KO*^ and patient-derived DRG neurons to qPCR and RNA-seq. Lowering the expression of the mutated allele (by KO) resulted in the recovery of previously altered gene expression, of genes related to neural induction (i.e., *MAP2*, *PAX6*) (Figures 2B and S2C), and of genes that were previously shown to be implicated by GNAO1 malfunction (*TUBB3*, *ANK3*).^38^ Moreover, GNAO1^iso-WT/KO^ NPCs form spheres in a size and shape similar to those of WT1 and WT2 NPCs, as compared to the oversized spheres observed in patient-derived NPCs (Figure 2C). When comparing the transcriptional signature of DRG neurons differentiated from all three cell lines, *GNAO1*^*iso-WT/KO*^ has a higher similarity to WT1 cells than to patient cells (Figures 2D and S2D). These findings suggest that reduction of the mutated *GNAO1* allele has a possible beneficial effect.

### Screening allele-specific ASOs targeting c.736_738delinsAAA

**Figure 3:**
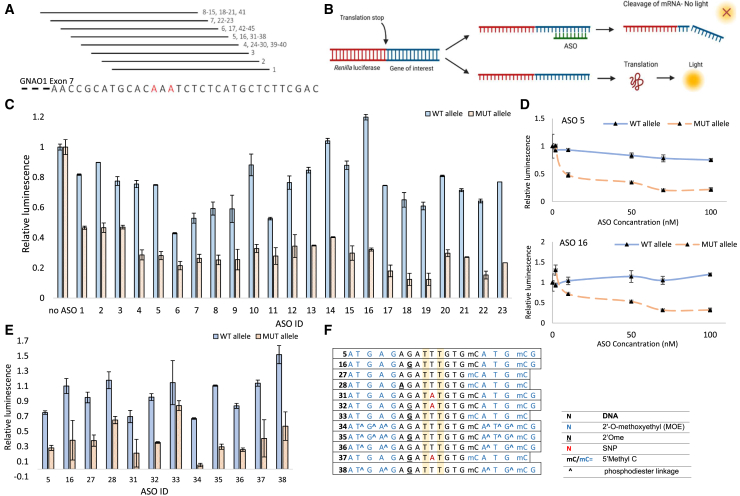
Screening allele-specific ASOs targeting the mutated GNAO1 allele (A) ASO library design scheme. Numbers on the right represent ASO-IDs (see Table 2). (B) Representative scheme showing the mechanism of action for the psi-CHECK system, utilized for synthetic ASO screen. (C) ASO-screen utilizing psi-CHECK platform. Luciferase levels for WT and MUT plasmids following ASO treatment (100 nM ASO, co-transfected with MUT or WT psi-CHECK plasmids). (D) Dose-response (10–100 nM) curves for ASO5 and ASO16 utilizing psi-CHECK platform. (E) psi-CHECK screen for optimized ASOs (100 nM) based on ASO5 and ASO16 sequences. (F) Partial list of personalized ASOs used in the work (sequences and chemistries). All ASOs include a full PS backbone, unless indicated otherwise. The full list can be found in Table 2.

We set out to screen multiple allele-specific ASOs aiming to selectively downregulate the expression of the mutated *GNAO1* allele. To that end, we designed a library of 19- to 20-nt PS-MOE ASOs (PS backbone with flanking 2′-MOE residues in a gapmer structure), targeting the sequence in which the pathogenic variant occurred, to achieve allele specificity^39^ (Figure 3A). The patient-unique sequence, including an additional SNV in close proximity to the pathogenic variant (Figure 1B), allowed better discrimination between the WT and Mutated alleles. ASOs differed in their sequence, length (9–10 DNA bases in the gap) and additional modification, such as 2′-*O*-methyl at specific positions, aiming to reduce potential toxicity^40^ (see Table 2). We added an additional mismatch to some of the sequences to further increase the difference between the WT and mutated alleles so as to increase specificity.^41^ To test the efficiency of these ASOs, we first employed a dual-luciferase reporter platform (psi-CHECK) containing either the WT sequence or the c.736_738delinsAAA sequence (referred to as WT or Mut plasmids) (Figure 3B). Each psi-CHECK plasmid was co-transfected with different ASOs, and luciferase intensity was quantified 48 h after transfection. ASOs showed different reduction efficiencies, between 50% and 90% of the Mut plasmid expression, with dramatic discrimination between the WT and Mut plasmids reaching 2- to 6-fold (Figures 3C and S3D). We further validated this effect using dose-response experiments, in which WT or Mut psi-CHECK transfected cells were subjected to increasing concentrations of the ASOs (Figure 3D). Lead candidates were defined as ASOs, which were both efficient and selective—significantly reducing the Mut levels (as measured by the luciferase reporter)—with minimal effect on the WT expression. New and optimized ASOs were screened utilizing the same platform (Figures 3E, 3F, and S3A–S3C). In some of the new ASOs, we used a mixed backbone, in which some of the PS bonds in the flanking regions were replaced with phosphodiester bonds, a structure that was shown to reduce neurotoxicity.^42^ The half-maximal inhibitory concentration was calculated for lead candidates (ASO16, -39, -41, and -44), and values showed dramatic differences between WT and Mut, starting from a 15-fold difference (ASO41, Mut: 10.36, WT: 136.5) and going up to a 50-fold difference (ASO39, Mut: 44.50, WT: 2,253) (Figure S3E). Overall, we screened 45 ASOs, out of which 12 ASOs showed satisfying results in terms of efficiency (50%–90% reduction of the Mut plasmid following 100-nM ASO treatment) and specificity (up to 10%–30% reduction of WT plasmid).

### Allele-specific ASOs selectively reduce mutated GNAO1 in patient-derived NPCs and improve patient-derived neuron functionality

**Figure 4:**
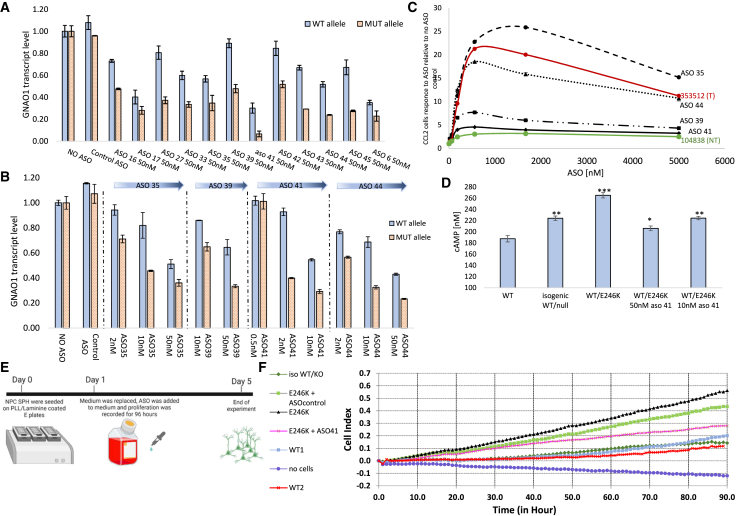
Testing allele-selective ASO on patient-derived DRG neurons (A) *GNAO1* WT and mutant transcript quantification using qPCR in patient-derived DRG neurons, gymnotically treated with different ASOs for 72 h. (B) *GNAO1* WT and mutant transcript levels of patient-derived DRG neurons gymnotically treated with different ASOs in increased concentrations for 72 h. (C) Toxicity prediction using qPCR of *CCL22* in BJAB cells gymnotically treated with increased concentrations of ASOs. ASO ISIS353512 was used as a toxic control (red), and ASO ISIS104838 was used as a non-toxic control (green). *CCL22* levels are presented as fold-increase comparing to non-treated cells (D) cAMP quantification in DRG neurons, derived from WT1, patient (E246K), and isogenic WT/KO iPSC. All cells were treated as indicated for 3 days. Following 1 μM forskolin induction for 20 min, cAMP level was determined using cAMP-Glo assay (∗*p* < 0.01; ∗∗*p* < 10^−5^; ∗∗∗*p* < 10^−7^). (E) Experiment scheme for proliferation assay using an RTCA system. (F) Proliferation (cell index) of two WT cell lines (WT1 and WT2), patient-derived (E246K), and isogenic WT/KO DRG neurons using E plate and RTCA system. Patient-derived cells were gymnotically treated with ASO41 or ASO control, and cells were monitored for 90 h (*n* = 5).

After screening 45 ASOs using the dual-luciferase reporter platform (Figure 3), we authentically tested lead candidates using patient-derived cells. Since *GNAO1* is predominantly active in neural lineage, we differentiated patient-derived iPSC to NPCs and DRG neurons (Figure S1B), which were subsequently gymnotically treated with varying concentrations of different ASOs. Both the efficacy and the selectivity of *GNAO1* reduction was evaluated using allele-discriminating primers, and qPCR allowed us to separately quantify the mutated and the WT alleles (Figure S4A). Lead ASOs showed significant reduction of the mutated allele, with a minimal decrease in the WT allele expression, tested following a 72-h treatment by gymontically delivered ASOs (60%–75% specific reduction) (Figures 4A, 4B, and S4B). Results obtained using qPCR in patient-derived NPCs were highly correlated with psi-CHECK results (Spearman’s *r* coefficient = 0.68, *p* < 0.0001) (Figure S4C). Importantly, these results were validated further by targeted RNA-seq conducted on patient-derived DRG neurons to validate qPCR screening results and to exclude possible artifacts or bias due to PCR conditions and primers (Figure S4D). Thus, we concluded that ASO35, ASO39, ASO41, and ASO44 showed the best results in terms of specificity and efficiency and subjected them to an *in vitro* toxicity prediction assay, focusing on potential innate immune system activation. To that end, we treated BJAB cells with increasing concentrations (20 nM– 5 μM) of the selected ASOs and measured innate immune activity based on *CCL22* expression by qPCR, as previously described.^43^^,^^44^ *CCL22* levels in the presence of lead ASOs were compared to a known toxic ASO (ISIS353512) and non-toxic ASO (ISIS104838), which served as a positive and negative controls, respectively.^45^^,^^46^ ASO41, ASO44, and ASO39 resulted in lower *CCL22* levels (comparing to the positive control) in all tested concentrations (Figure 4C). Potential off-targets were suggested by the alignment of the chosen sequences using BLAST and PFRED,^47^ revealing four annotated genes with a sequence homology of 15 or 16 bases (out of 19–20). Off-target effects were evaluated using RNA-seq for ASO-treated neurons, harvested after a 72-h treatment, reveling no reduction in transcript levels of said potential off-targets (Figure S4E). Next, we set out to find whether ASO treatment can have a functional effect on patient-derived cells. Since *GNAO1* is known to affect cAMP levels, we used a cAMP quantification assay (cAMP-Glo assay) to test the effect of ASO treatment in patient-derived DRG neurons. Each cell line was treated with an activating agent (forskolin) and cAMP hydrolysis inhibitor (IBMX), and cAMP levels were quantified. Untreated patient-derived cells showed higher levels of cAMP compared to WT cells, as seen in different platforms described in previous publications.^1^^,^^5^ This accumulation was resolved in cells treated with ASO (Figure 4D) and in GNAO1^iso-WT/KO^ cells, thus emphasizing the potential therapeutic effect of such interventions. We next utilized live-proliferation tracking to examine the proliferation rate of patient-derived DRG neurons at days 1–5 (untreated, gymnotically treated with 50 nM ASO41 or control ASO) compared to that of WT DRGs and GNAO1^iso-WT/KO^ (Figure 4E). As expected, untreated patient-derived neurons showed a much higher proliferation rate than both WT and GNAO1^iso-WT/KO^ (Figure 4F). ASO41 treatment has significantly decreased the proliferation of cells during the 90-h experiment.

### Gnao1^E246K^ mouse model shows a neurological phenotype

**Figure 5:**
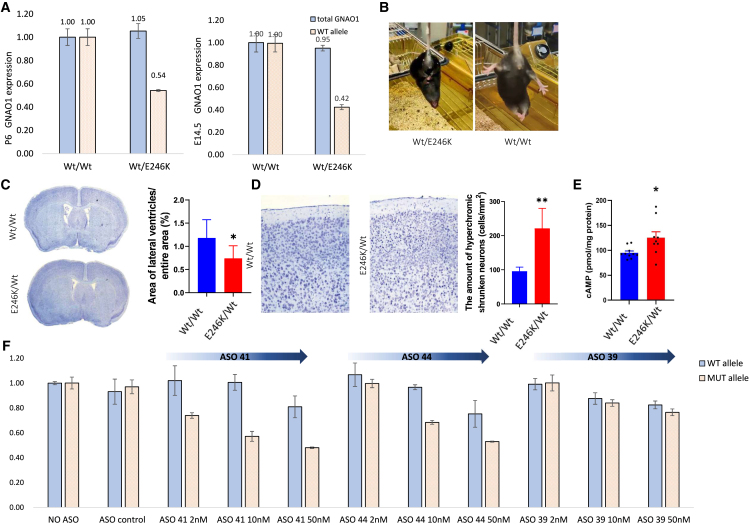
Gnao1^E246K^ mouse model (A) Total and WT *GNAO1* transcript level in E14 and P6 mouse brains (*n* = 4). (B) Representative picture of Gnao1^Wt/E246K^ mouse showing a typical neurological symptom- limbs clasping induced by tail suspension. (C and D) Effects of the E246K mutation on brain morphology were examined by analyzing coronal brain sections from E246K/+ and WT^+/+^) littermates. Nissl staining showed a reduction in lateral ventricles. Morphometric analysis revealed an increased number of hyperchromatic neurons in the motor cortex of the mutant mice (n = 3, ∗*p* < 0.05; ∗∗*p* < 0.01). (E) ELISA determination of total cAMP in striatal tissue from E246K/+ mice and WT^+/+^ littermates, harvested at P8 (*n* = 9/10 per group) (nonparametric t test; Mann-Whitney test, *p* = 0.0206). (F) *Gnao1* WT and mutant allele transcript levels following ASO gymnotic treatment in neurons differentiated from E14.5 Gnao1^Wt/E246K^ brains (*n* = 3).

To further decipher the therapeutic opportunity presented herein, we established a mouse model with a corresponding mutation in *mGnao1*, using CRISPR-Cas9 zygote injection. The sequence in which the patient’s pathogenic variant occurred is highly conserved between human and mouse, allowing us to generate a mutated murine copy of *Gnao1* with the exact same genomic sequence as the patient (Figures S5A and S5B). Gnao1^Wt/E246K^, our newly established knockin mice carrying the E246K mutation (C57BL/6), includes both the pathogenic single-nucleotide variant (SNV) and the additional synonymous SNV identified in the patient’s DNA (Figures S5A and S5B). RNA derived from NPCs isolated from the embryonic day 14.5 (E14.5) Gnao1^Wt/E246K^ embryos and brains of postnatal day 6 (P6) mice show that overall RNA levels of *Gnao1* are similar to those of WT littermates (Gnao1^Wt/Wt^) (Figure 5A). RNA-seq of brains harvested from P21 heterozygote mice show that the mutated allele is expressed (Figure S5C).

The heterozygote mice are viable and fertile and do not show any visible abnormal features or behavior during the postnatal period and adolescence. At the age of 3 months, approximately 25%–30% of the heterozygotes develop neurological symptoms (Figures 5B and S5D), either spontaneously or in response to mild stress (routine handling). The spectrum of neurological responses includes limb clasping induced by tail suspension (Figure 5B), dystonic postures, seizures (lasting approximately 30 s) and abnormal motor behavior (slow recovery from stress, temporary lack of movement) (Video S1). More than 20% mice experience spontaneous seizures and/or dystonic attacks, and all male mice showing dystonic attacks in response to the tail suspension test also exhibit premature death within a short time from the first symptom (Figure S5D).

Video S1. Gnao1-E246K mice experiencing spontaneous or stress-induced seizures A 4-months old heterozygote male

Previous research has demonstrated that point pathogenic variants in the GNAO1 gene can lead to impaired development of the cerebral cortex in patients with GNAO1 mutations^6^^,^^13^^,^^26^^,^^27^^,^^48^ and in animal models.^18^ To investigate the effects of E246K on the brain structure, we analyzed coronal brain sections from heterozygous (Gnao1^Wt/E246K^) and WT littermates using the Nissl staining method (Figure 5C). We measured the areas of brain slices, lateral ventricles, and motor cortex thickness in the regions of interest (Figures 5C and S5E). Our analysis revealed a significant reduction in the area of brain slices and lateral ventricles in mutant mice compared with WT (Figures 5C and S5E), whereas there was no significant difference in the thickness of the motor cortex between the two groups (Figure S5E). The brain slices from mutant mice also showed a higher number of hyperchromatic neurons (Figure 5D). This phenomenon may be related to cytoskeletal retraction,^49^ which has been observed in animal models of epilepsy. cAMP levels in Gnao1^Wt/E246K^ was measured utilizing a biochemical ELISA-based approach.^1^ Significantly higher cAMP levels were detected in striatal tissues collected from P8 heterozygote pups (Gnao1^Wt/E246K^) compared with WT^+/+^ littermates (Figure 5E).

To validate the compatibility of our ASOs with the mouse sequence, we derived and cultured NPCs from E14.5 *Gnao1*^*Wt/E246K*^ embryos. Cultured NPCs were treated for 48 h with gymnotically delivered ASOs (ASO41/ASO44/ASO39, 2–50 nM), and analysis showed selective and dose-dependent reduction of the mutated allele (Figure 5F). In agreement with the patient-derived cellular model, ASO41 shows the highest efficiency, with a 24%–54% decrease in the mutated allele, with almost no effect on the WT allele.

## Discussion

During the last few years, RNA therapy has dramatically progressed, with a consistent increase in approved drugs and clinical trials.^20^ Different modalities, especially ASO, were also recruited for personalized medicine, taking advantage of the versatility, rationale design, and proven safety of these drugs. ASO drugs are also well suited to treating the CNS^50^ due to their excellent bio-distribution following intrathecal or intracerebroventricular injections^50^^,^^51^; thus, they have an advantage over other modalities and approaches, in which the access to the brain is limited by the blood-brain barrier, or toxic responses. In this work, we explore a possible beneficial effect for a patient harboring a dominant *GNAO1* variant by selective reduction of the mutated allele, using allele-specific ASO.

An initial library of ASOs was first tested on the 293T cell line, utilizing a dual-luciferase reporter plasmid, containing either the WT or the mutated sequence (Figure 3). ASOs differed in their sequence, although all versions contained the mutation site, aiming to achieve allele specificity. Additional changes were applied to the different ASOs, including various chemistries, modification of gap length, and additional mismatches. A shorter gap (9 nt) correlated with higher specificity and higher efficiency, as seen in previous works^52^^,^^53^ (Figure S3D). Additional mismatches added to the sequences improved specificity to the mutated allele, but had reduced efficiency. Another aspect that had a large impact on both specificity and efficiency of the ASO is the location of the variant in the ASO. Specific positioning of the differential bases in the gap (namely closer to the 5′ flanking wing), contributed to increased specificity, due to the RNAse-H1 cleavage pattern.^53^^,^^54^

We then proceeded to test our lead candidates using patient-derived NPCs and DRG neurons (Figure 4). psi-CHECK results and results from the cellular model were highly correlated, encouraging our decision to move forward with specific candidates based on the preliminary screen. Treatment functionality was evaluated using two important parameters distinguishing between patient-derived cells and WT, the first one being proliferation. When establishing the patient-derived cellular model, we noticed an accelerated proliferation rate, which was seen in both iPSCs and NPCs. The effect of Gαo activity and expression on proliferation was discussed in previous publications, especially regarding different malignancies.^55^^,^^56^ Another paper showed that a somatic mutation in Gαo, R209C (a known germline hotspot pathogenic variant linked to a neurodevelopmental disorder), can stimulate cell proliferation and even contribute to neoplastic events, resulting in acute lymphoblastic leukemia.^57^ Furthermore, murine *Gnao1* was described as a regulator of Schwann cell proliferation.^58^ While it is important to emphasize that no correlation was shown between disease-causing *GNAO1*-germline pathogenic variants and malignancies, these notions suggest an effect of *GNAO1* on cell proliferation. As our notions were consistent with these publications, we used proliferation rate as a readout and were able to reduce it both by knocking out the mutated allele and by treating the patient’s cells with allele-specific ASOs. We also tested the effect of ASOs on cAMP levels in patient-derived DRG neurons, showing that the treatment can resolve the accumulation of cAMP seen in untreated cells. Lead candidates were also subjected to toxicity prediction assays to eliminate poorly tolerated ASOs, leaving us with two to three leading candidates that excelled in all tests so far.

As adequate animal models are fundamental and necessary for understating the disease pathophysiology, *GNAO1* has been modeled so far in *Drosophila*,^59^ *C. elegans*,^60^ and mice.^1^^,^^18^^,^^61^^,^^62^ These models showed different extents of phenotypic recapitulation, all contributing to further deciphering of the disease and testing of potential therapeutics.^63^ To the best of our knowledge, our work was the first to establish and characterize a *Gnao1-E246K* mouse model. Previous publications reported mouse models for other patients’ hotspot pathogenic variants in *GNAO1*, including G203R, C215Y,^18^ and R209H,^61^ as well as one activating mutation that was not identified in patients (G184S).^62^ These mice show a variety of phenotypes, the most severe one seen in G203R, which showed dramatically reduced viability.^18^ Both C215Y and R209H heterozygote mice are hyperactive, as shown in several behavioral tests, including open field test and swimming behavior (swimming tank test). Our work on the Gnao1^E246K^ mouse model has not been extended to behavioral tests. Given the model’s apparent neurological symptoms, which include spontaneous or stress-induced dystonic episodes and seizures, it would be interesting to see whether it also shows a behavioral phenotype. As *GNAO1* is highly conserved, the specific sequence of interest shows complete homology between human and mouse. Thus, we were able to test our ASOs, designed specifically for the patient, using our mouse model, as shown by using cultured NPCs from the brains of the E14.5 E246K heterozygote embryos, treated with varying concentrations of ASOs. Analysis showed dosage-dependent reduction in mutated *Gnao1*, as seen in patient-derived DRG neurons, proving that our model is amenable for treatment with the personalized ASO.

In summary, we report the identification of a potent and discriminative allele-specific ASO, aiming to selectively reduce the *GNAO1* mutated allele (c.736_738delinsAAA). While the work focuses on E246K, the same approach may be relevant for additional *GNAO1* pathogenic variants.^64^ Personalized treatments using RNA therapy was proven to be possible, with the first drug approved for treatment in a single patient, Milasen,^65^ given to a patient suffering from Batten disease. Milasen was followed by the research and development of additional n of 1 customized ASOs for different conditions^42^^,^^66^^,^^67^ under a supportive and progressing regulatory environment,^67^^,^^68^ thus enabling and encouraging additional research groups to take part in this new form of personalized medicine.^69^ We hope to translate our findings on *GNAO1* allele-selective targeting toward the clinic and to utilize personalized ASOs to give hope to patients with *GNAO1*-related disorders.

## Materials and methods

### Reprogramming of fibroblasts to iPSCs

Patient fibroblasts were derived from a skin-punch biopsy (institutional review board [IRB] approval no. 6158-19-SMC). Next, fibroblasts were reprogrammed to iPSCs utilizing the StemRNA 3rd Gen Reprogramming Kit (Stemgent). Briefly, fibroblasts were seeded at a density of 7.5 × 10^4^ cells per well in 6-well plates. Cells were transfected four times by a RNA reprogramming cocktail. iPSC colonies appeared approximately 4 days after the last transfection. The appearing iPSC colonies were manually picked and expended on Geltrex (Gibco)-coated 6-well plates. Splitting and re-plating of iPSCs were achieved by detachment using Versene (Gibco) and seeding in Nutristem medium (Sartorius). iPSCs were analyzed by qPCR for *OCT4*, *NANOG*, and *LIN28* pluripotency markers and by alkaline phosphatase detection kit (Millipore). Normal human donor iPSCs (KYOU-DXR0109B) were purchased from American Type Culture Collection (WT1) or reprogrammed from healthy donor fibroblasts (IRB 6158-19-SMC) (WT2).

### Differentiation of iPSCs to DRG neurons and ASO treatment

iPSCs were differentiated to neurons as described previously by Goldstein et al.^31^ In brief, iPSCs were grown on Geltrex-coated 6-well plates in Nutristem medium until they reached 80% confluence. Cells were detached using Versene and seeded in 24 hollow agar molds at a density of 400,000 cells per mold. Cells were grown in MI medium containing Glasgow’s MEM, 10% KO replacement serum, 1% l-glutamine, 1% pyruvate, 1% non-essential amino acids, 0.1 μM β-mercaptoethanol, and 1% PSA Antibiotic-Antimycotic solution (Gibco). During the first 4 days, the cells were supplemented with 20 μM dorsomorphin (Tocris), 10 μM SB431542 (Miltenyi Biotec), and 10 μM Rho kinase inhibitor (Enzo Life Sciences). A total of 14 days from the initial seeding, embryoid bodies were formed and seeded on poly-d-lysine/laminin (Sigma) 24-well plates. The neurons were further differentiated for 7–14 days in DRG medium containing DMEM/F12.2% B27 (Gibco) and 10 ng/μL of nerve growth factor, neurotrophin-3, glial cell line-derived neurotrophic factor, and brain-derived neurotrophic factor (Alomone). Medium was replaced three times per week. DRG neurons were analyzed by RT-qPCR for *bTub3*, *MAP2*, *NSE*, and *PAX6*, and stained for Tuj (R&D Systems, MAB1195) and neurofilament heavy polypeptide (Abcam, ab8135). For ASO screens on neurons gymnotic uptake was applied and followed by RNA extraction at day 7.

### RT-qPCR

RNA isolation of iPSC-derived neurons was performed using ReliaPrep RNA Cell Miniprep System (Promega), according to the manufacturer’s instructions. Isolated RNA (1,000 ng) of each sample was reverse transcribed to cDNA by using the High-Capacity cDNA RT Synthesis Kit (Applied Biosystems). Real-time quantitative PCR was performed on the BioRad CFX96 system in technical triplicate per sample by adding 5 μL cDNA (5 ng/μL) and primer pairs (listed in Table 1) to SYBR Green Master Mix (Applied Biosystems). *GAPDH* was used as a housekeeping gene, and relative quantification (RQ) values (RQmin/RQmax) were determined using the CFX Maestro system. RT-qPCR for differentiation markers and GNAO1 levels was conducted for four independent differentiation experiments, each including three biological repeats, all quantified in technical triplicate.

**Table 1:** List of primers used in this study

hOCT4 Fw	GCTCGAGAAGGATGTGGTCC	real-Time PCR
hOCT4 Rv	CGTTGTGCATAGTCGCTGCT	real-Time PCR
hNANOG fw	GCAGAAGGCCTCAGCACCTA	real-Time PCR
hNANOG rv	AGGTTCCCAGTCGGGTTCA	real-Time PCR
Human GAPDH fw	CTCCTGCACCACCAACTGCT	real-Time PCR
Human GAPDH rv	GGGCCATCCACAGTCTTCTG	real-Time PCR
hTubb3 Fw	AACCAGATCGGGGCCAAGTT	real-Time PCR
hTubb3 Rv	AGGCACGTACTTGTGA GAAGAG	real-Time PCR
hPAX6 Fw	CTGAGGAATCAGAGAA GACAGGC	real-Time PCR
hPAX6 Rv	ATGGAGCCAGATGTGA AGGAGG	real-Time PCR
hMAP2 Fw	AGGCTGTAGCAGTCCT GAAAGG	real-Time PCR
hMAP2 Rv	CTTCCTCCACTGTGACA GTCTG	real-Time PCR
hLIN28 Fw	*GAAGCGCAGATCAAAAGGAG*	real-Time PCR
hLIN28 Rv	*GCTGATGCTCTGGCAGAAGT*	real-Time PCR
hNSE Fw	GGAACTGCCCCTGTATCGCC	real-Time PCR
hNSE Rv	CTGCACCTAGTCGCATGGCA	real-Time PCR
Mouse GAPDH F	AATGTGTCCGTCGTGGATCT	real-Time PCR
Mouse GAPDH R	AGACAACCTGGTCCTCAGTG	real-Time PCR
h/mGNAO1mutAD_Fw	CGAACCGCATGCACAAA	real-Time PCR
hGNAO1 bothAD_Rv	GATCTTCTCGCCAAAGAGAT	real-Time PCR
h/mGNAO1WTAD _Fw	CGAACCGCATGCACGAG	real-Time PCR
mGNAO1 both AD Rv	GCAGATGGTCAAGGGT GACTTC	real-Time PCR

### ASO design

The ASOs used in this study were synthesized by Ella Biotech (PS-MOE ASOs) and included PS backbone modifications and flanking 2-MOE residues in a gapmer structure. ASOs were diluted in deuterium-depleted water (stock: 100 μM) and used as indicated for each experiment. A total of 45 ASOs were used for specific mutant allele silencing (Table 2).

**Table 2:** List of ASOs used in this study

Reference sequence	AACCGCATGCACAAATCTCTCATGCTCT
No.	Sequence
1	eG∗eA∗eG∗Z∗eA∗T∗G∗A∗G∗A∗G∗A∗T∗T∗T∗eG∗eT∗eG∗Z∗eA
2	eA∗eG∗Z∗eA∗eT∗G∗A∗G∗A∗G∗A∗T∗T∗T∗G∗eT∗eG∗Z∗eA∗eT
3	eG∗Z∗eA∗eT∗eG∗A∗G∗A∗G∗A∗T∗T∗T∗G∗T∗eG∗Z∗eA∗eT∗eG
4	Z∗eA∗eT∗eG∗eA∗G∗A∗G∗A∗T∗T∗T∗G∗T∗G∗Z∗eA∗eT∗eG∗Z
5	eA∗eT∗eG∗eA∗eG∗A∗G∗A∗T∗T∗T∗G∗T∗G∗mC∗eA∗eT∗eG∗Z∗eG
6	eT∗eG∗eA∗eG∗eA∗G∗A∗T∗T∗T∗G∗T∗G∗mC∗A∗eT∗eG∗Z∗eG∗eG
7	eG∗eA∗eG∗eA∗eG∗A∗T∗T∗T∗G∗T∗G∗mC∗A∗T∗eG∗Z∗eG∗eG∗eT
8	eA∗eG∗eA∗eG∗eA∗T∗T∗T∗G∗T∗G∗mC∗A∗T∗G∗Z∗eG∗eG∗eT∗eT
9	eA∗eG∗eA∗eG∗eA∗T∗T∗T∗G∗T∗G∗mC∗A∗T∗T∗Z∗eG∗eG∗eT∗eT
10	eA∗eG∗eA∗eG∗eA∗T∗T∗T∗G∗T∗G∗mC∗A∗A∗T∗Z∗eG∗eG∗eT∗eT
11	eA∗eG∗eA∗eG∗eA∗T∗T∗T∗G∗T∗G∗mC∗A∗A∗G∗Z∗eG∗eG∗eT∗eT
12	eA∗eG∗eA∗eG∗eA∗T∗oT∗T∗G∗T∗G∗mC∗A∗T∗G∗Z∗eG∗eG∗eT∗eT
13	eA∗eG∗eA∗eG∗eA∗T∗oT∗T∗G∗T∗G∗mC∗A∗T∗T∗Z∗eG∗eG∗eT∗eT
14	eA∗eG∗eA∗eG∗eA∗T∗oT∗T∗G∗T∗G∗mC∗A∗A∗T∗Z∗eG∗eG∗eT∗eT
15	eA∗eG∗eA∗eG∗eA∗T∗oT∗T∗G∗T∗G∗mC∗A∗A∗G∗Z∗eG∗eG∗eT∗eT
16	eA∗eT∗eG∗eA∗eG∗A∗oG∗A∗T∗T∗T∗G∗T∗G∗mC∗eA∗eT∗eG∗Z∗eG
17	eT∗eG∗eA∗eG∗eA∗G∗oA∗T∗T∗T∗G∗T∗G∗mC∗A∗eT∗eG∗Z∗eG∗eG
18	eA∗eG∗eA∗eG∗eA∗T∗T∗T∗G∗T∗G∗mC∗A∗T∗eG∗Z∗eG∗eG∗eT
19	eA∗eG∗eA∗eG∗eA∗T∗T∗T∗G∗T∗G∗mC∗A∗A∗eG∗Z∗eG∗eG∗eT
20	eA∗eG∗eA∗eG∗eA∗T∗oT∗T∗G∗T∗G∗mC∗A∗T∗eG∗Z∗eG∗eG∗eT
21	eA∗eG∗eA∗eG∗eA∗T∗T∗T∗G∗T∗G∗mC∗A∗A∗eG∗Z∗eG∗eG∗eT
22	eG∗eA∗eG∗eA∗eG∗A∗T∗T∗T∗G∗T∗G∗mC∗A∗eT∗eG∗Z∗eG∗eG
23	eG∗eA∗eG∗eA∗eG∗A∗T∗T∗T∗G∗T∗G∗mC∗T∗eT∗eG∗Z∗eG∗eG
24	Z∗eA∗eT∗eG∗eA∗G∗oA∗G∗A∗T∗T∗T∗G∗T∗G∗Z∗eA∗eT∗eG∗Z
25	Z∗eA∗eT∗eG∗eA∗G∗oA∗mC∗A∗T∗T∗T∗G∗T∗G∗Z∗eA∗eT∗eG∗Z
26	Z∗eA∗eT∗eG∗eA∗G∗A∗G∗A∗T∗A∗T∗G∗T∗G∗Z∗eA∗eT∗eG∗Z
27	eA∗eT∗eG∗eA∗eG∗A∗G∗A∗T∗T∗T∗G∗T∗G∗Z∗eA∗eT∗eG∗Z
28	eA∗eT∗eG∗eA∗eG∗A∗oG∗A∗T∗T∗T∗G∗T∗G∗Z∗eA∗eT∗eG∗Z
29	Z∗eAˆeTˆeGˆeA∗G∗A∗G∗A∗T∗T∗T∗G∗T∗G∗ZˆeAˆeTˆeG∗Z
30	Z∗eAˆeT∗eGˆeA∗G∗A∗G∗A∗T∗T∗T∗G∗T∗G∗ZˆeA∗eTˆeG∗Z
31	eA∗eT∗eG∗eA∗eG∗A∗G∗A∗T∗A∗T∗G∗T∗G∗mC∗eA∗eT∗eG∗Z∗eG
32	eA∗eT∗eG∗eA∗eG∗A∗oG∗A∗T∗A∗T∗G∗T∗G∗mC∗eA∗eT∗eG∗Z∗eG
33	eA∗eT∗eG∗eA∗eG∗A∗oG∗A∗T∗T∗T∗G∗T∗G∗Z∗eA∗eT∗eG∗Z
34	eA∗eTˆeGˆeAˆeG∗A∗G∗A∗T∗T∗T∗G∗T∗G∗mC∗eAˆeTˆeGˆZ∗eG
35	eA∗eTˆeGˆeAˆeG∗A∗oG∗A∗T∗T∗T∗G∗T∗G∗mC∗eAˆeTˆeGˆZ∗eG
36	eA∗eTˆeG∗eAˆeG∗A∗G∗A∗T∗T∗T∗G∗T∗G∗mC∗eAˆeT∗eGˆZ∗eG
37	eA∗eT∗eG∗eA∗eG∗A∗oG∗A∗T∗A∗T∗G∗T∗G∗Z∗eA∗eT∗eG∗Z
38	eA∗eTˆeG∗eAˆeG∗A∗oG∗A∗T∗T∗T∗G∗T∗G∗mC∗eAˆeT∗eGˆZ∗eG
39	Z∗eA∗eT∗eG∗eA∗oG∗A∗G∗A∗T∗T∗T∗G∗T∗eG∗Z∗eA∗eT∗eG
40	Z∗eA∗eT∗eG∗eA∗G∗A∗G∗A∗T∗T∗T∗G∗T∗eG∗Z∗eA∗eT∗eG
41	eA∗eG∗eA∗eG∗eA∗oT∗T∗T∗G∗T∗G∗mC∗A∗T∗eG∗Z∗eG∗eG∗eT
42	eT∗eG∗eA∗eG∗eA∗G∗A∗T∗T∗T∗G∗T∗G∗mC∗eA∗eT∗eG∗Z∗eG
43	eT∗eG∗eA∗eG∗eA∗oG∗A∗T∗T∗T∗G∗T∗G∗mC∗eA∗eT∗eG∗Z∗eG
44	eT∗eGˆeA∗eGˆeA∗oG∗A∗T∗T∗T∗G∗T∗G∗mC∗eAˆeT∗eGˆZ∗eG
45	eT∗eGˆeAˆeGˆeA∗oG∗A∗T∗T∗T∗G∗T∗G∗mC∗eAˆeTˆeGˆZ∗eG
Control	eG∗eT∗eG∗eA∗eG∗G∗G∗mC∗A∗G∗T∗A∗A∗A∗A∗eA∗eA∗eA∗eT∗eA
N	DNA
eN	2′-*O*-methoxyethyl (2′-MOE)
oN	2′-Ome
mC/Z	5′-methyl C/2′-MOE 5′-methyl C
∗	phosphorothioate linkage
ˆ	phosphodiester linkage

### Immunocytochemistry

iPSC-derived neurons were fixated on day 7 of DRG differentiation with 4% paraformaldehyde (PFA; Electron Microscopy Sciences) for 15 min and subsequently washed 3 times with PBS. Cells were permeabilized with 0.25% Triton X-100 (Sigma) for 15 min and washed with PBS; subsequently, blocking was performed with 1% BSA and 2% serum in PBS with Tween 20 for 30 min. Fixed and permeabilized neurons were incubated with Tuj (R&D Systems, MAB1195) and neurofilamentH (Abcam, ab8135, ) 1:500 in 1% BSA for 1 h at room temperature. After an additional washing step, cells were incubated with secondary antibody 1:1,000 (Abcam, ab150077) in 1% BSA for 1 h. DAPI (Sigma, D9542) was used to visualize the nucleus, and images were acquired using a ZOE microscope.

### psiCHECK

psiCHECK-2 vector was purchased from Promega. A 30-bp fragment of GNAO1 flanking the mutation site was introduced to the vector by PCR and fused to synthetic Renilla luciferase reporter gene. This vector possesses a secondary firefly reporter expression cassette to normalize the relative plasmid quantity.

GNAO1 WT fragment: GAACCGCATGCACGAGTCTCTCATGCTCTT

GNAO1 mutant E246K fragment: GAACCGCATGCACAAATCTCTCATGCTCTT

The 293T cells were seeded at density of 7 × 10^4^ per well in a white 96-culture plate. The assay was performed in triplicate. Each well was transfected using Lipofectamine 3000 (Thermo Fisher), with 150 ng WT or mutant GNAO1 pSI-CHECK plasmid and control or GNAO1 ASO in the indicated concentrations. Medium was replaced the following day, and luminescence was measured 2 days following transfection using the Dual-Glo Luciferase assay system (Promega). Relative luminescence was calculated as the ratio of renilla to firefly luciferase in each well. The normalized read was calculated as the ratio of relative luminescence in treated vs. control (untreated) wells.

### cAMP measurements

DRG neurons were seeded on poly-d-lysine/laminin-treated 24-well plates. Four days post-seeding, DRG neuron medium was replaced and cells were gymnotically treated with GNAO1 or control ASO in indicated concentrations. Three days post-transfection, cells were treated with 100 μM IBMX (Tocris, 2845) to inhibit cAMP hydrolysis and 1 μM forskolin (Sigma, F3917) to stimulate adenylate cyclase. cAMP-Glo assay (Promega) or competitive cAMP ELISA (Abcam) was performed in four replicates per treatment to measure the cAMP level. cAMP produced following receptor-independent activation was calculated as the delta of forskolin-treated to untreated cells’ luminescence.

### Proliferation assay

Following 14 days of neuronal induction in MI medium, neuronal NPCs were transferred from agar molds to poly-d-lysine/laminin-coated E plates (ACEA Biosciences). Two NPC spheres were placed in each well, with each cell line seeded in triplicates. Cells were allowed to adjust overnight and were gymnotically treated with control or GNAO1 ASO for 96 h. During that time, the cell index was recorded by a real-time cell analysis dual-purpose (RTCA DP) instrument.

### Isogenic WT/KO cells

Guide RNA (gRNA; GCATGAGAGATTTGTGCATGCGG) was cloned into the pKLV-gRNA vector. Next, patient-derived iPSCs underwent co-Electroporation with CAS9 expressing plasmid, pKLV-gRNA and homology-directed repair (HDR) sequence (CTAATTCTCTCCTTCTCTTTCCCTGTCTCTGTGTCTCCCTCCCGCTGTCTGTCCTCTCTCCTCCCTTCCTGCGGCCCGAGAATAGGATGCACGAGTCTCTCATGCTCTTCGACTCCATCTGTAACAACAAGTTCTTCATCGATACCTCCATCATTCTCTTCCTCAACAAGAAA). Puromycin and hygromycin were added to the medium to eliminate untransfected cells, and the remaining colonies were analyzed using PCR for the specific HDR sequence. The genomic sequence was validated by Sanger sequencing and later by RNA-seq, as described.

### Poly(A) RNA-seq

RNA was extracted from DRG neurons (day 7) or NPCs as described previously. mRNA was isolated using NEBNext Poly(A) mRNA Magnetic Isolation Module (NEB) followed by NEBNext Ultra II Directional RNA Library Prep Kit for Illumina (NEB) for library preparation. Libraries were sequenced on an Illumina NovaSeq 6000 sequencer (Illumina) to generate paired-end (100-bp) reads for each sample. RNA-seq read quality was evaluated using FastQC, and adapter sequences were removed with Trim Galore. Reads were aligned to the human genome (GRCh38) using STAR followed by PCR duplicate removal by the unique molecular identifiers. Alignment quality was evaluated using Picard Tools. HTseq-count was used to count the number of reads for each gene after alignment. Differential expression analysis was performed with the DEseq2 package in R. GO term enrichment analysis was performed using the clusterProfiler package in R. The heatmap was generated using EnhancedVolcano and pheatmap packages, respectively.

### GNAO1 targeted RNA-seq

RNA was extracted from DRG day 7 neurons and reverse transcribed to cDNA, as described previously. Next, 250-bp fragments of GNAO1 and HS2ST1 (as normalizing control) were amplified by PCR (18 cycles with Q5 High-Fidelity 2X Master Mix [NEB]) (primers are listed in Table 3). Nextera transposase adapters were added during this process (marked by an underline). Fragments were cleaned by Agencourt AMPure XP beads and followed by 11 cycles with Nextera XT Index Primer (NEB) and KAPA HiFi HotStart ReadyMix (Roche). Libraries were sequenced on an Illumina MiSeq System to generate 250-bp reads for each sample. Reads were aligned against GNAO1 and HS2ST1 amplicon references using Bowtie2. SAMtools Pileup was used to generate the combined nucleotide frequency for each position, which was parsed using an in-house script.

**Table 3:** List of primers used for GNAO1-targeted RNA-seq library

Fw HS2ST1nextera	TCGTCGGCAGCGTCAGATGTGTATAAGAGACAGCAGAGAAGCTCTGGCTTCA
Rv HS2ST1 nextera	GTCTCGTGGGCTCGGAGATGTGTATAAGAGACAGGAATTGGAACTGCTCTAGTG
Fw GNAO1nextera	TCGTCGGCAGCGTCAGATGTGTATAAGAGACAGGCGATCTGAACGCAAGAAG
Rv GNAO1nextera	GTCTCGTGGGCTCGGAGATGTGTATAAGAGACAGGATGGTCAAAGGTGACTTC

### Toxicity and BJAB cells

BJAB cells were used to test potential toxicity of different ASOs using a protocol described in Pollak et al.^44^ Briefly, human BJAB cells were cultured in suspension at 37°C in RPMI medium (15% serum, 1% penicillin-streptomycin, 1% glutamine, and 1% sodium pyruvate). Cells were quantified and diluted to 200,000 cells in 24-well plates before beginning the experiment. After plating, cells were treated with different ASOs at different concentrations (20 nM–5 μM), including positive control (ISIS353512), incubated for 16 h at 37°C, and subsequently harvested. After RNA purification, human *CCL22* was quantified with qPCR using the following primers: forward: 5′-CGCGTGGTGAAACACTTCTA-3′, reverse: 5′-GATCGGCACAGATCTCCTTATC-3′.

### E246K mouse model

Mouse blastocyte injection was approved by the Weizmann Institute’s Institutional Animal Care and Use Committee (IACUC) and were carried out in accordance with their approved guidelines. gRNAs were designed using CRISPOR.^70^

A single gRNA was designed to target gene Gnao1 (5′-GCATGAGAGACTCGTGCATG-3′). A single-stranded oligodeoxynucleotide (ssODN) donor repair template flanked asymmetrically by homology arms to each of the 5′ and 3′ insertion sites was designed (5′-GTGTGTGTCTCTCTCTGTCTTGCTCTCTCCCCTCCCCGCCGGGGCTGCAGAATCGCATGCACAAATCTCTCATGCTCTTCGACTCCATCTGTAACAACAAGTTTTTCATTGATACCTCCATCATC-3′). Cas9 nuclease, CRISPR RNA, *trans*-activating CRISPR RNA, and ssODN were purchased from Integrated DNA Technologies.

Genetically modified mGnao1^E246K^ mice were generated at the transgenic facility at the Weizmann Institute of Science using CRISPR-Cas9 genome editing in isolated one-cell mouse embryos as described.^71^ C57Bl/6JOlaHsd mice were purchased from Envigo and maintained in specific pathogen-free conditions. Mice were maintained on a 12-h light/dark cycle, and food and water were provided *ad libitum*. Cas9-gRNA ribonucleoprotein complexes together with a donor repair template were delivered to one-cell embryos via electroporation, using the Bio-Rad Gene Pulser. Electroporated embryos were transferred into the oviducts of pseudopregnant ICR female mice (Envigo). Genomic DNA from F0 pups was analyzed at weaning by PCR and Sanger sequencing using primers: forward: 5′-TTGGCACAGAATGGTGGATA-3′, reverse: 5′-GCAGATGGTCAAGGGTGACT-3′.

All subsequent animal experiments described here were approved by the Sheba Medical Center IACUC (1306/21/ANIM, 0025/22/ANIM), and the colony was maintained in the Sheba animal facility. Mice were genotyped using the sequencing reverse primer (5′-GCAGATGGTCAAGGGTGACT-3′), and two forward primers (WT: 5′-CCGCATGCACGAGTCTCTC-3′, mutant: 5′-GCTGCAGAATCGCATGCACAAA-3′).

### Tail suspension

To test the susceptibility of the mice to dystonic episodes, a blinded tail suspension test was conducted. Mice were picked up by the tail and suspended for 30 s 15–20 cm above the floor of the cage and then placed in a clean cage. Mice were observed, and any abnormal clasping of the hindlimbs was indicated as a phenotypic event.

### Measurement of Brain cAMP

Brain cAMP levels were measured as previously described.^1^ Briefly, striatal tissues were harvested from Gnao1^E246K^^^/+^^ P8 pups and WT^+/+^ littermates. Tissue dissection was performed on ice. Collected tissues were flash frozen in liquid nitrogen and followed by homogenization in 0.1 M HCl. cAMP levels were determined by diluting the samples (between 1:20 and 1:50) in 0.1 M HCl, followed by quantification with a competitive cAMP enzyme immunoassay, following the acetylated protocol described in the manufacturer’s guidelines (Direct cAMP ELISA Kit, Enzo Life Sciences). Extrapolated cAMP values were normalized to protein concentration (QPRO-BCA Kit Standard, Cyanagen).

### Embryonic NPC culture

Cortices of E13 or E14 embryos were dissected mechanically and immediately plated in low-adherence plates in NeuroBasal media containing B27 (×1) (Gibco), GlutaMAX (x1) (Gibco), gentamicin (25 μg/mL) (Tocris), epidermal growth factor (20 ng/mL) (Tocris), basic fibroblast growth factor (20 ng/mL) (Tocris), and heparin (2 μg/mL) (STEMCELL Technologies). To obtain adherent Neurospheres, cells were plated on 24-well poly-l-lysine/laminin-coated plates in growth factor-depleted media. NPC neurons were gymnotically treated the next day with ASOs, as described, followed by RNA extraction 48 h post-transfection. WT and mutant allele frequency was determined by RT-qPCR, as previously described.

### Histological analysis and immunohistochemistry

Brains were collected from 12-week-old mice, fixed in 4% PFA, embedded in paraffin, and cut into 5-μm sections. The sections were mounted on glass slides and stored at 4°C until use. For morphometric analysis, identified sagittal sections were prepared from WT^+/+^; *n* = 3) and E246K/+ (*n* = 3) mice. The sections were deparaffinized, rehydrated, and stained by the Nissl method. At least two serial sections from three different animals for each genotype were photographed using an Axio-Scan.Z1 slide scanner (Carl Zeiss). All neurons visible in all layers were evaluated according to their chromatophilia (the intensity of staining of neuron cytoplasm) and classified into normochromic (normal or medium staining) and hyperchromic (intense staining) or hyperchromic shrunken cells. Image analysis was carried out using NIH ImageJ software. The following parameters were measured: area of brain slices, cortex thickness (perpendicular to the lateral ventricular region), and area of the lateral ventricles. To achieve consistent comparisons across the groups, three mice from each genotype were analyzed using the same criteria and techniques.

## Data and code availability

The authors confirm that the most relevant data supporting the findings of this study are available within the article and/or its supplemental information. Additional data analyzed in the study are available from the corresponding authors on reasonable request.

## Acknowledgments

We thank Ms. Golda Damari, Dr. Alina Berkovitz, and Ms. Sima Peretz of the Weizmann Institute’s Transgenic and Knockout Core Facility for help in preparing the mouse model used in this study. We also thank Dr. Elena Ainbinder of the Weizmann Institute’s stem cells core facility for her help in establishing the genetically modified iPSC line used in this study. This work was supported by 10.13039/501100006769Russian Science Foundation grant no. 21-15-00138 to D.N.S. and V.L.K. to contribute to the brain anatomy investigation of the mutant mice. The authors would like to thank the patient’s family for their valuable cooperation.

## Author contributions

I.S., N.M. and D.D. conceived the idea and wrote the manuscript, with significant contributions from other team members. I.S. performed the cell culture and molecular experiments. S.R., N.B.-H., and A.H. performed the *in vivo* experiments. T.M. performed the toxicity prediction experiments. A.Z. assisted in the cell culture. R.F. performed the bioinformatics analysis. R.H.-K. generated the mouse model, based on Y.R.’s design. S.A.-N. assisted in ASO and design of the experiments. D.N.S. and V.L.K. performed the investigational anatomy analysis of the mouse model. B.B.-Z. and G.H. helped with clinical interpretation.

## Declaration of interests

The authors declare no competing interests.

## Supplemental information

Document S1. Figures S1–S5

Document S2. Article plus supplemental information
