# Supplementary material for: Personalized allele-specific antisense oligonucleotides for GNAO1-neurodevelopmental disorder
Source: Mol Ther Nucleic Acids. 2024 Dec 22;36(1):102432. doi: 10.1016/j.omtn.2024.102432 (PMC11787015; doi:10.1016/j.omtn.2024.102432)
Supplement: Document S1. Figures S1–S5 [file mmc1.pdf]

OMTN, Volume *36*

Supplemental information

Personalized allele-specific antisense oligonucleotides for GNAO1-neurodevelopmental disorder

Inna Shomer, Nofar Mor, Shaul Raviv, Noga Budick-Harmelin, Tanya Matchevich, Sharon Avkin-Nachum, Yoach Rais, Rebecca Haffner-Krausz, Ariela Haimovich, Aviv Ziv, Reut Fluss, Bruria Ben-Ze'ev, Gali Heimer, Denis N. Silachev, Vladimir L. Katanaev, and Dan Dominissini

Supplemental Figures

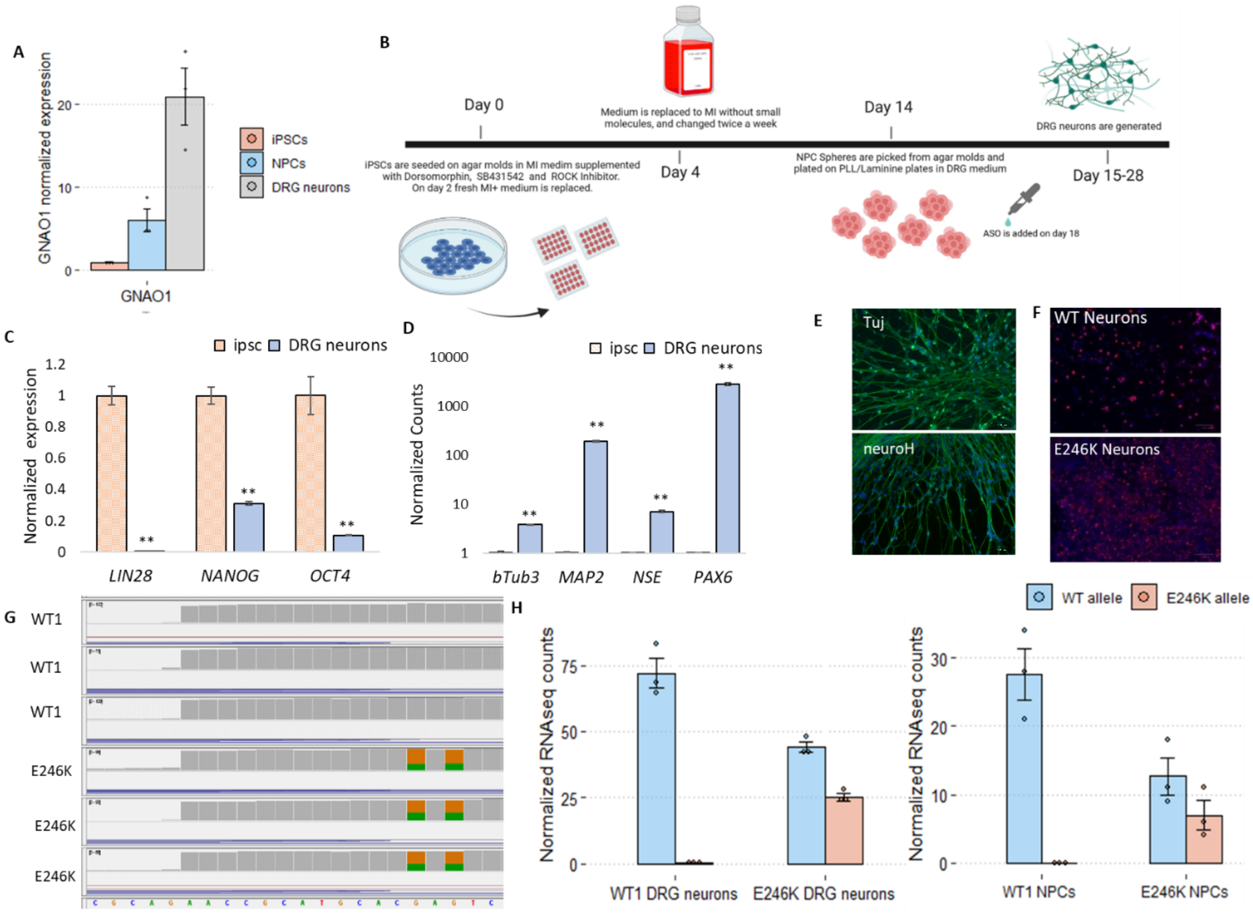

Figure S1. Establishing neuronal differentiation system and using it to evaluate phenotype and GNAO1 level expression in patient-derived neurons. (A) Relative *GNAO1* transcript level in iPSCs, neuro-progenitor cells (NPCs) and DRG neurons (day 7), quantified by rtPCR. (B) Schematic experimental scheme of neuronal differentiation from patient derived iPSC. (C) Transcripts' levels of pluripotency markers (normalized to *GAPDH)* ** p-value< 0.001 (D) RNA-seq normalized counts for neuronal markers in iPSC-derived neurons** p-value< 0.001 (E) Immunostaining of WT1 iPSC-derived neurons for neuronal markers Tuj and neuro-filamentH (neuroH). (F) Ki-67 staining of WT1 comparing to patient-derived (E246K) DRG neurons. (G) *GNAO1* reads from WT1 and patient (E246K) DRG neurons, sequenced in RNA-seq. Orange-G bases, Green- A bases. (H) Quantification of *GNAO1* WT and E246K transcript reads in WT1 or patient-derived neuro-progenitor cells (NPCs) and DRG neurons (day 7) following RNA-seq analysis.

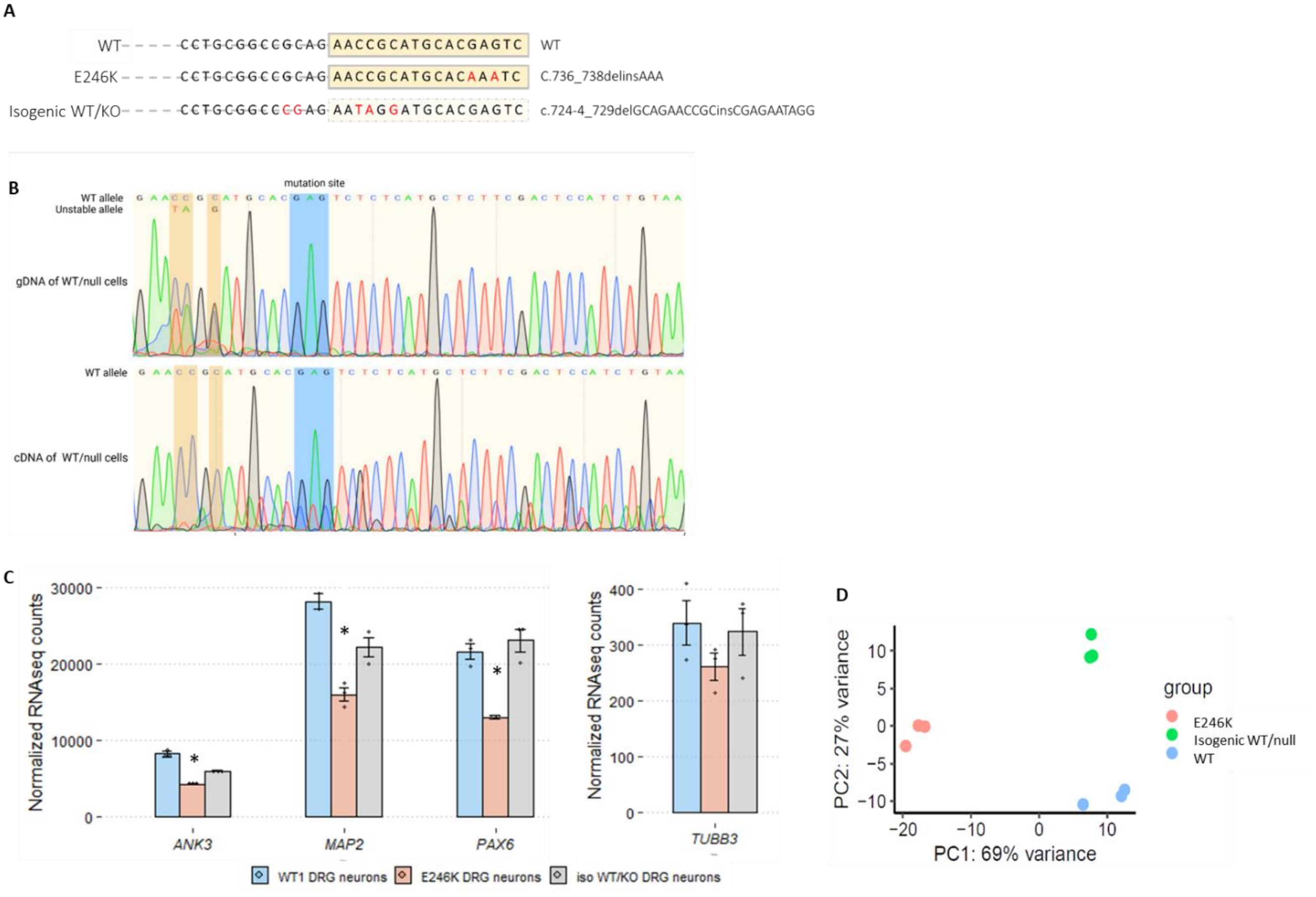

Figure S2. Establishing isogenic GNAO1 heterozygote iPSCs (A) Schematic representation of isogenic GNAO1 WT/KO iPSC sequence design – red letters in the lower panel (isogenic WT/KO cell line) represent mutations introduced by HDR correction. The box represents the exon. (B) Sanger chromatograms for genomic DNA (gDNA) and cDNA (represents RNA) of WT/KO cells, showing the altered allele present in the gDNA and absent as RNA. (C) RNA-seq analysis of neuronal differentiation markers *MAP2*,*PAX6*, *bTUBB3* and *ANK3* in WT1, patient (E246K) and isogenic WT/KO iPSC-derived DRG neurons (* p value<0.01), n=3. *bTUBB3* is not significant. (D) RNA-seq based PCA plot of iPSC-derived DRG neuron

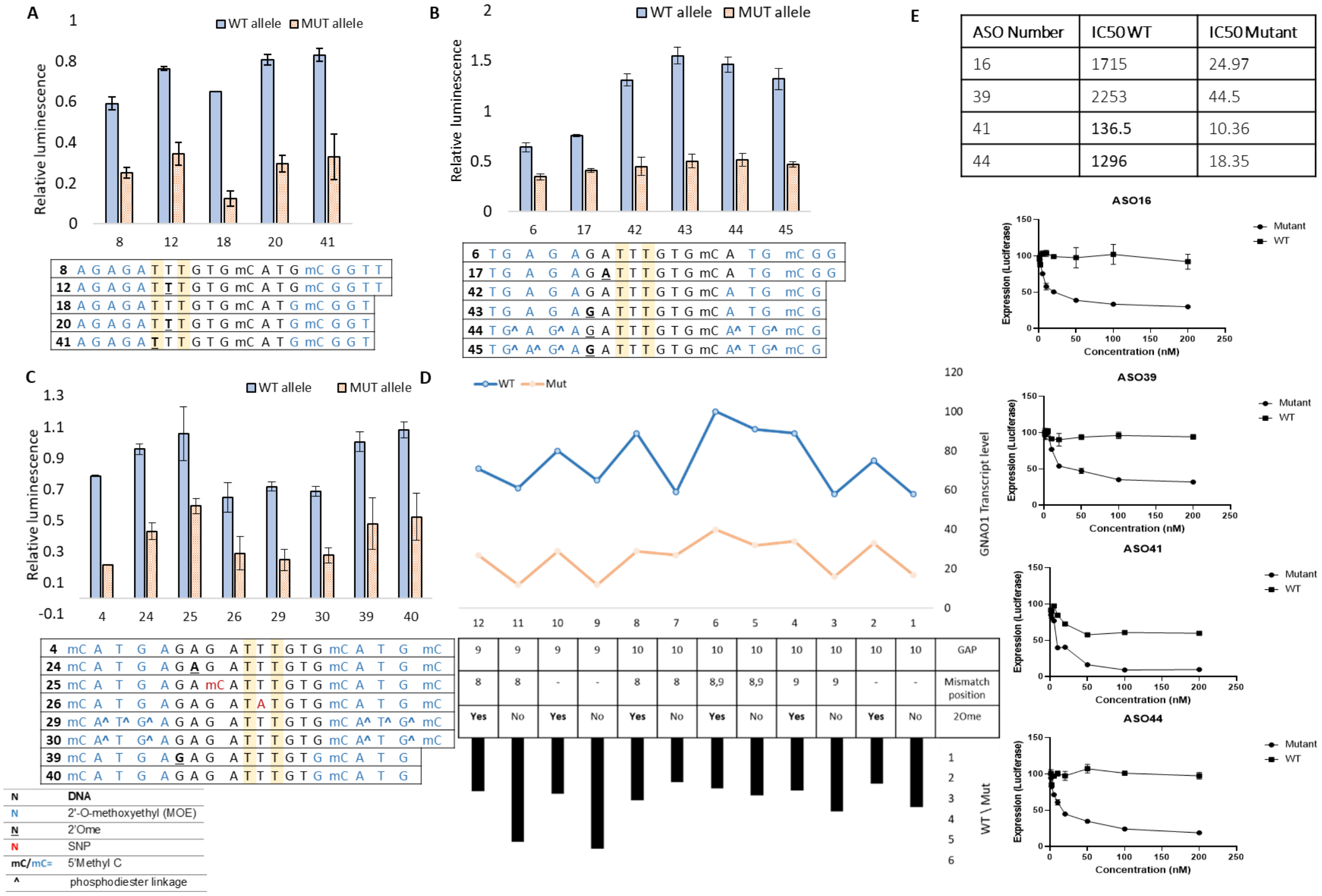

ASO Number	IC50 WT	IC50 Mutant
16	1715	24.97
39	2253	44.5
41	136.5	10.36
44	1296	18.35

Figure S3. Screening Allele-specific ASOs targeting mutant GNAO1 allele. (A,B,C) Reduction of WT and MUT plasmids (psi-CHECK) following ASO treatments (100nM), quantified by luminescence levels (n=3) (D) Diagram representing ASO efficacy and specificity to target sequence, based on psi-CHECK screen. Mutation positioning is the same in all presented ASOs, and different properties of the ASO are examined, including gap length (9/10 bp), additional mismatch (position, if any), and additive 2'-Ome. (E) IC50 of different ASOs for WT and Mutant *GNAO1* in the psi-CHECK platform.

A

GNAO1mutAD_Fw (Forward MUT specific primer)	CGAACCGCATGCACAAA
GNAO1wtAD_Fw (Forward WT specific primer)	CGAACCGCATGCACGAG
GNAO1 bothAD_Rv (Reverse to WT and MUT alleles)	GATCTTCTCGCCAAAGAGAT

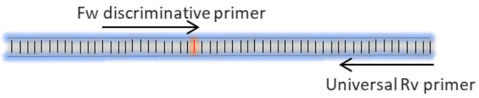

C

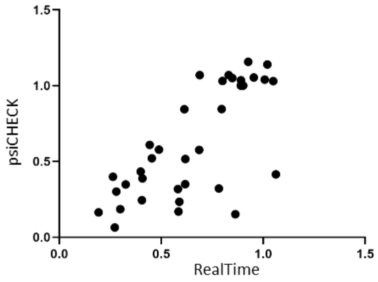

B

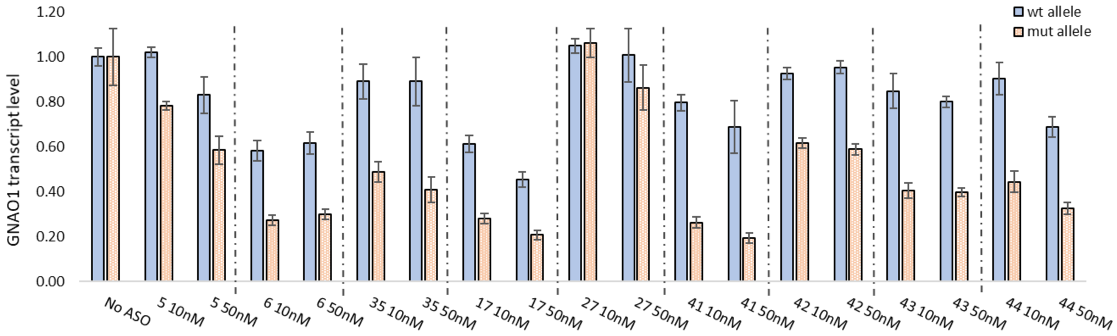

D

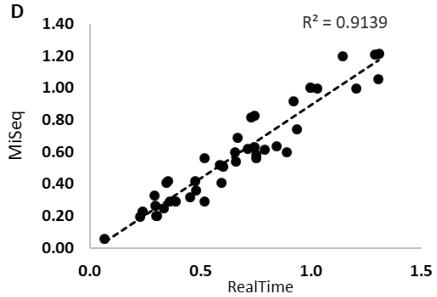

E

	Alignment	41 (2nM)	44 (5nM)
ASTN2	16/16	NS	NS
GDAP1	16/16	NS	NS
MME	15/15	NS	NS
USH2A	15/15	NS	NS

Figure S4. Testing allele-specific ASO on patient-derived DRG neurons. (A) Primer design for allele discriminating rtPCR. (B) *GNAO1* WT and mutant alleles' transcript level of patient-derived DRG neurons gymnotically treated with ASOs in two concentrations for 72 hours. n=3 (C) Correlation of relative Mutant to WT allele quantification (Mut\WT) following different ASOs treatment, comparing rtPCR (patient-derived DRG neurons) and psi-CHECK (overexpressed in HEK293T). R (Spearman) = 0.68, p value<0.0001. (D) Correlation between relative Mutant to WT allele quantification (Mut\WT), comparing rtPCR analysis and NGS analysis (RNA-seq targeted library) R (Spearman) = 0.9139 (E) RNA-seq based transcript analysis of potential off-targets in ASO-treated comparing to untreated patient-derived DRG (NS= Non significant)

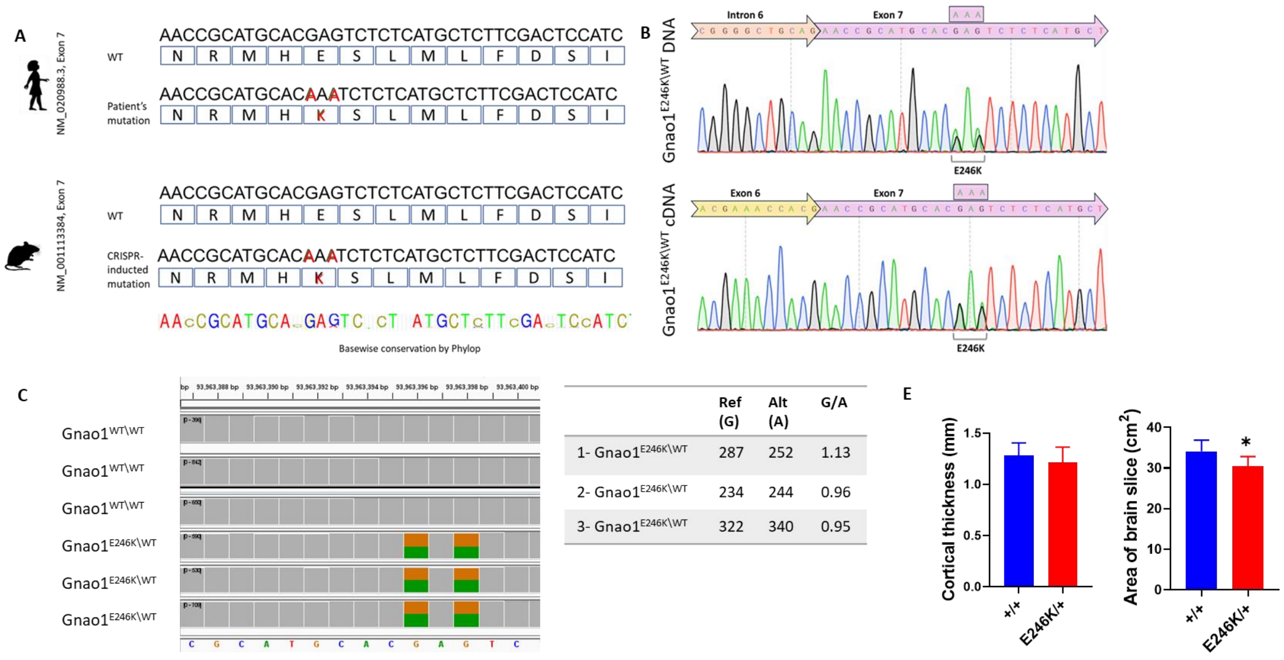

D

	Total number of heterozygous mice	Spontaneous phenotype				Tail suspension test				
		Mice showing phenotype		Premature death of mice showing phenotype (between 4-9 months)		Total number of tested heterozygous mice	Mice showing phenotype		Premature death of mice showing phenotype (between 4-7 months)	
		Number of mice	%	Number of mice	%		Number of mice	%	Number of mice	%
**Males and Females**	**91**	**19**	**20.9**	**4**	**21.1**	**54**	**3**	**5.5**	**3**	**100**
Males	41	7	17.1	3	42.9	27	3	11	3	100
Females	50	12	24.0	1	8.3	27	0	0		

Figure S5. Gnao1^{E246K} mouse model **(**A) Genomic sequences of human and mouse *GNAO1*, mutations (patient in human and CRISPR-induced in mouse) are marked in red. The genomic area in which the patient's mutation occurred is evolutionary conserved, as shown by PhyloP multiple alignments of 100 vertebrate species. (B) Sanger sequencing chromatograms for gDNA and cDNA heterozygote E246K mouse brains harvested at day 21 (C) Mutant and WT alleles' levels in heterozygote E246K mice brains (quantified by RNA-seq analysis). Orange-G bases, Green- A bases.(D) Quantification of spontaneous and induced neurological phenotypes seen in heterozygote E246K mice. (E) The effects of the E246K mutation on brain morphology were examined by analyzing coronal brain sections from E246K/ + and wild-type (+ / +) littermates. Nissl staining showed a reduction in the area of brain slices in the mutant mice, but no significant changes in cortical thickness (N=3, $p < 0.05$ (*)).

Video S1 – Gnao1-E246K mice experiencing spontaneous or stress induced seizures. A 4-months old heterozygote male (Gnao1$^{WT/E246K}$) suffering a seizure following tail-suspension.
